# Low-FODMAP formula improves diarrhea and nutritional status in hospitalized patients receiving enteral nutrition: a randomized, multicenter, double-blind clinical trial

**DOI:** 10.1186/s12937-015-0106-0

**Published:** 2015-11-03

**Authors:** So Ra Yoon, Jong Hwa Lee, Jae Hyang Lee, Ga Yoon Na, Kyun-Hee Lee, Yoon-Bok Lee, Gu-Hun Jung, Oh Yoen Kim

**Affiliations:** 1 Department of Food Science Nutrition, Dong-A University, Brain Busan 21 Project, Busan, 604-714 Republic of Korea; 2 Department of Rehabilitation Medicine, Dong-A University Hospital, Busan, South Korea; 3 Central Research Institute, Dr. Chung’s Foods Co., Ltd., Cheongju, Chungbuk Republic of Korea

**Keywords:** Poorly absorbed, Short-chain carbohydrates, Enteral nutrition, Diarrhea, Prealbumin, Transferrin, body mass index

## Abstract

**Background:**

Fermentable oligosaccharides, disaccharides, monosaccharides, and polyols (FODMAPs) are poorly absorbed, short-chain carbohydrates that play an important role in inducing functional gut symptoms. A low-FODMAP diet improves abdominal symptoms in patients with inflammatory bowel disease and irritable bowel syndrome. However, there were no study for the effect of FODMAP content on gastrointestinal intolerance and nutritional status in patients receiving enteral nutrition (EN).

**Methods:**

In this randomized, multicenter, double-blind, 14-day clinical trial, eligible hospitalized patients receiving EN (*n* = 100) were randomly assigned to three groups; 84 patients completed the trial (low-FODMAP EN, *n* = 30; moderate-FODMAP EN, *n* = 28; high-FODMAP EN, *n* = 26). Anthropometric and biochemical parameters were measured; stool assessment was performed using the King’s Stool Chart and clinical definition.

**Results:**

Baseline values were not significantly different among the three groups. After the 14-day intervention, diarrhea significantly improved in the low-FODMAP group than in the moderate- and high-FODMAP groups (*P* < 0.05). King’s Stool scores in diarrhea subjects were significantly and steadily reduced in the low-FODMAP group compared with the other two groups (*P* for time and EN type interaction <0.05). BMI increased significantly in the low- and high-FODMAP groups during the intervention (*P* < 0.05 for both), and showed a trend toward increasing in the moderate-FODMAP group (*P* < 0.10). Serum prealbumin increased significantly in all groups by 14-day; by 3-day, it had increased to the levels at 14-day in the low-FODMAP group. At 14-day, serum transferrin had increased significantly in the moderate-FODMAP group. In addition, subjects were classified by final condition (unimproved, normal maintenance, diarrhea only improved, constipation only improved, and recurrent diarrhea/constipation improved). Seventy-five percent of the diarrhea improved group consumed the low-FODMAP EN formula. 38.5 and 46.2 % of recurrent diarrhea/constipation improved group consumed the low- and moderate-FODMAP EN respectively. BMI significantly increased in all groups except the unimproved. Prealbumin levels significantly increased in the diarrhea-improved and recurrent diarrhea/constipation groups at 3-day and continued by 14-day, and in the constipation-improved group at 14-day. Transferrin levels significantly increased in the diarrhea-improved and recurrent diarrhea/constipation groups at 14-day.

**Conclusion:**

Low-FODMAP EN may improve diarrhea, leading to improved nutritional status and facilitating prompt recovery from illness.

**Electronic supplementary material:**

The online version of this article (doi:10.1186/s12937-015-0106-0) contains supplementary material, which is available to authorized users.

## Background

Enteral nutrition (EN) is a common method of nutrition support for hospitalized patients with intact gastrointestinal function who are unable to eat or whose nutritional requirements are not satisfied with an oral diet [1]. Despite the benefits of EN (e.g., shortening the length of hospital stays and lowering rates of infectious complications, readmissions, and mortality) [2–4], gastrointestinal symptoms, such as diarrhea, constipation, bloating, abdominal pain, flatulence, and vomiting/nausea, frequently occur [5]. These symptoms may cause malnutrition (i.e., imbalanced fluid and electrolytes and/or inadequate intake/absorption of nutrients), thereby preventing prompt recovery from illness [6, 7]. Diarrhea, in particular, is a frequently observed gastrointestinal symptom in patients receiving EN [8–10]: it occurs in anywhere of hospitalized EN patients, depending on their health [11–13]. The cause of EN-associated diarrhea is unclear, but likely multifactorial. Absorption problems, feeding temperature, feeding method, and the high osmolality and nutrient composition of EN formulas are probable factors [14–19]. Many have endeavored to solve the problem by changing feeding method, temperature, or formula content [6], but the results have been inconsistent.

Recently, it has been suggested that diarrhea is associated with fermentable oligosaccharides, disaccharides, monosaccharides, and polyols (FODMAPs) which is poorly absorbed, short-chain carbohydrates [8, 9, 20–23]. FODMAPs are found in a wide variety of foods, including apples, mangos, and fructose syrup (fructose); onions, garlic, and rye (fructans); milk (lactose); legumes (galactans); and mushrooms, stone fruit, and some artificial sweeteners (polyols) [24]. They are poorly absorbed in the small intestine and, when delivered to the colon, may produce gas and subsequently cause luminal distension, disturb gut motility, and result in diarrhea [23–25]. A low-FODMAP diet significantly reduces gastrointestinal symptoms in patients with irritable bowel syndrome (IBS) and inflammatory bowel disease (IBD) [20–23, 26]. Of note, low-FODMAP dietary interventions improve gastrointestinal symptoms more in IBS patients with fructose malabsorption problems than in those without [22]. A retrospective EN study reported that the rate of diarrhea development was lower in patients given a lower-FODMAP formula than those given other types of formulas [8]: although length of hospital stay and duration of EN independently predicted diarrhea development, this study suggested that being provided with a lower-FODMAP formula could potentially mitigate these risks [8, 9].

To date, all the intervention studies for the association between gastrointestinal symptom and FODMAPs were performed in the patients with IBS or IBD, however there has been no prospective intervention study investigating the effect of FODMAP content on diarrhea and nutritional status in patients receiving EN. Therefore, this randomized, double-blind 14-day clinical trial investigated the effect of FODMAP content in EN formula on gastrointestinal intolerance and nutritional status in hospitalized patients receiving EN.

## Materials and methods

### Study design and participants

This study was a randomized, multicenter, double-blind, 14-day clinical trial. Between November 2013 and November 2014, participants were recruited from 10 hospitals located in Busan and its environs, in the Republic of Korea. Five were university hospitals (Dong-A University Hospital, Busan National University Hospital and Dong-Eui Medical Center in Busan, and Busan National University Hospital in Yangsan) and six were rehabilitation and long-term care hospitals (Bumin Hospital, Centum Erooda Hospital, Happy Medical Center, Inchang Hospital, Keunsol Medical Hospital, and Medwill hospital in Busan). All participants aged 20 years or over were hospitalized and receiving EN. Exclusion criteria included the following: 1) EN forbidden due to ileus, gastrectomy, bleeding in the digestive tract, etc.; 2) renal or liver dysfunction, e.g., renal failure, nephritis, nephrotic syndrome, hepatitis, cirrhosis, serum creatinine > 1.5 mg/dl, serum urea nitrogen > 25 mg/ml, serum aspartate aminotransferase > 40U/L, or serum alanine aminotransferase > 40U/L; 3) uncontrollable diabetes; and 4) pregnancy or breast-feeding. All study participants (or their legal guardians) were provided with detailed information about the study and provided written informed consent. One hundred sixty five subjects were assessed for eligibility. One-hundred eligible subjects were randomly assigned to low-FODMAP EN, moderate-FODMAP EN, or high-FODMAP EN for 14 days using a block randomization method. The patients, clinicians and all study personnel were blinded to the contents of the experimental EN formulas. The experimental EN formulas were indistinguishable color and provided in identical cans. Allocation concealment was maintained using a centralized, web-based randomization schedule accessible 24 h a day. Energy needs were estimated by a registered dietitian (RD) based on the patient’s height, weight and disease condition. Basically, patients received intermittent nasogastric EN feeding (100–400 ml/20–40 min, 3 times/day). To avoid unexpected gastrointestinal problems due to a sudden change in EN formula, each patient basically had a 3-day adaptation period in which one-third the volume of the former formula was replaced with the new, experimental EN formula each day. Sixteen participants dropped out during the intervention period, leaving 84 to complete the study. Of the 16 dropouts, 2 were transferred to another hospital, 2 stopped EN and changed to a transitional diet, 4 severe diarrheas and 8 withdrew for personal reasons. Finally, 84 patients completed the trial (low-FODMAP EN, *n* = 30; moderate-FODMAP EN, *n* = 28; high-FODMAP EN, *n* = 26) were included for analysis. The study protocol was approved by the Institutional Review Board of Dong-A University (the representative, approval no. 2-1040709-AB-N-01-201310-BR-03-03) and carried out in accordance with the Helsinki Declaration. The study’s clinicaltrial.gov identifier is NCT02353689.

### Nutrient composition of experimental enteral nutrition (EN) formulas

Three types of EN formula were provided by Dr. Chung’s Foods Co., Ltd. (Cheongju, Chungbuk, Republic of Korea); the FODMAP content and nutrient composition of each are described in Table 1. All the experimental EN formulas contained the same number of calories per can (200 kcal/200 ml), but different FODMAP contents (low-FODMAP: 0.320 g/can; moderate-FODMAP: 0.753 g/can; high-FODMAP: 1.222 g/can). Most of nutrient contents except some micronutrients are similar in all the EN formulas and they were formulated following to and within the guideline of Korean dietary reference intakes.

**Table 1:** FODMAP content and nutrient composition of experimental EN formulas

Per can (200 mL)	Low-FODMAP	Moderate-FODMAP	High-FODMAP
Calorie (kcal)	200	200	200
Total FODMAPs (g)	0.320	0.753	1.222
Fructose (g)	- (N.D.)	-	-
Lactose (g)	-	-	-
Raffinose (g)	0.079	0.229	0.285
Stachyose (g)	-	-	0.239
1-Kestose (g)	-	-	0.511
Nystose (g)	-	-	-
1-Fructofuranosylnystose (g)	0.233	0.509	0.411
Carbohydrates (g)	28	32	31
Sugar (g)	0	1	2
Fiber (g)	3	3	4.3
Protein (g)	9	8	9
Fat (g)	7	6	6
Sodium (mg)	135	180	135
Vitamin A (μgRE)	225	140	150
Vitamin B1 (mg)	0.24	0.3	0.24
Vitamin B2 (mg)	0.3	0.36	0.3
Vitamin B6 (mg)	0.3	0.45	0.3
Vitamin B12 (μg)	0.96	1	0.48
Vitamin C (mg)	40	30	20
Vitamin D (μg)	1	1	1
Vitamin E (mg α-TE)	4.8	5	2
Vitamin K (μg)	15	11	15
Folic acid (μg)	80	75	80
Niacin (mg NE)	3.2	2.6	3.2
Biotin (μg)	6	6	6
Pantothenic acid (mg)	1	1	1
Calcium (mg)	150	140	140
Phosphorus (mg)	140	140	140
Magnesium (mg)	58	22	58
Zinc (mg)	4	2.4	2
Iron (mg)	2	2.3	2
Potassium (mg)	225	308.5	220
Manganese (mg)	0.8	0.4	0.7
Copper (mg)	0.16	0.23	0.16
Iodine (mg)	30	23	30

*N.D*. non-detected, *NE* niacin equivalents, *α*-*TE* α-tocopherol

### Data collection

Baseline data included: patient demographics (age, gender, height and body weight), disease history, dietitian assessment of nutritional requirement etc. Data were collected daily for up to 14 days post-randomization for stool assessment, energy intake, time taken to reach 100 % experimental EN feeding. Anthropometric and biochemical parameters were also collected at 0-day (before starting), 3-day (4^th^ day morning) and 14-day (after the intervention) for the monitoring of nutritional status.

### Outcomes

The primary outcome was the improvement of diarrhea assessed by the King’s Stool chart and the clinical definition. Secondary outcomes were the levels of plasma prealbumin and transferrin, the markers for short-term nutritional status. Other outcomes were changes of anthropometric parameters, other biochemical parameters and other gastrointestinal symptoms.

### Stool assessment

Stool assessment data were collected daily for up to 14 days. It was performed using the King’s Stool Chart, which incorporates descriptors of stool frequency, weight, and consistency, as previously described [27–29]. The chart has been validated for use in free-living individuals, patients at high-risk of diarrhea and patients receiving enteral nutrition [27.28]. Briefly, the chart comprises three categories of stool weight (<100 g, 100–200 g, >200 g) and four categories of stool consistency (hard and formed, soft and formed, loose and unformed, liquid). This results in 12 possible combinations of stool weight and consistency, each accompanied by a verbal and pictorial descriptor to assist accurate characterization [27–29]. Each category is also assigned an alphabetical code, so that physicians, nurses, and dieticians can record and communicate fecal output using standard verbal and pictorial descriptors. Fecal frequency is incorporated by recording the code of each feces passed; each category is assigned a score, enabling the summation of all scores in a 24-h period to obtain a daily fecal score. In this study, diarrhea was classified as a daily fecal score of 15 or more. Diarrhea was also classified using a clinical definition: 1) unformed or liquid stools for two or more days; or 2) unformed and liquid stools (≥200 g) three times or more per day. Constipation was defined as a condition of fewer than three bowel movements per week or no defecation for three or more days, requiring stool softeners or an enema at least once.

### Anthropometric parameters, blood pressure, and blood collection

Height and body weight were measured when possible (62 of 84 patients). Body mass index (BMI) was calculated as body weight divided by height squared (kg/m^2^). Blood pressure (BP) was obtained from the left arm of seated individuals with an automatic BP monitor (HEM-7220, OMRON, Matsusaka, Japan) after a short rest. Triceps skin fold thickness (TSF, cm), midarm circumference (MAC, mm) and midarm muscle circumference (MAMC [cm] = TSF [cm] – MAC [cm] × 3.14) were also measured. Blood specimens were collected three times over the intervention period (0-day, 3-day and 14-day). After an overnight fast, venous blood specimens were collected in EDTA-treated or plain tubes. The tubes were immediately placed on ice until they arrived at the analytical laboratory (1–3 h). Blood specimens were then separated into plasma and serum and stored at −80 °C until analysis.

### Serum glucose, lipid profile, albumin, prealbumin, transferrin and blood cell counts

Fasting glucose was measured using a glucose oxidase method (Glucose Analyzer Beckman Instruments, Irvine, CA, USA), as described in a previous report [30]. Serum total cholesterol, triglycerides, and low-density lipoprotein (LDL) cholesterol levels were measured using commercially available kits on a Hitachi 7150 Auto-analyzer (Hitachi Ltd., Tokyo, Japan), as described previously [31]. After precipitating serum chylomicrons, LDL-cholesterol, and very low-density lipoprotein with dextran sulfate-magnesium, the high-density lipoprotein (HDL) cholesterol left in the supernatant was measured using a previously described enzymatic method [31]. Serum fasting albumin, preabumin and transferrin were measured using commercially available kits on a Hitachi 7150 Auto-analyzer (Hitachi Ltd., Tokyo, Japan). White blood cell (WBC) and absolute lymphocyte (ALC) counts were determined using the HORIBA ABX diagnostics system (HORIBA ABX SAS, Parc Euromedicine, France).

### Safety parameters: liver and kidney functions

Serum aspartate aminotransferase (AST) and alanine aminotransferase (ALT) were measured using a modified International Federation of Clinical Chemistry (IFCC) UV method. Serum creatinine was measured with a kinetic colorimetric (Jaffe) assay. Serum concentrations of blood urea nitrogen (BUN) and uric acid were measured using a kinetic UV Assay.

### Serum high-sensitivity C-reactive protein and plasma interleukin-6, interleukin-1β, and tumor necrosis factor-α

Serum high-sensitivity C-reactive protein (hs-CRP) was measured with an ADVIA 1650 system (Bayer, Tarrytown, NY, USA) using a commercially available, high-sensitivity CRP-Latex(II) *X*2 kit (Seiken Laboratories Ltd., Tokyo, Japan), which allows detection of CRP in the 0.001–31 mg/dL range, as described in a previous report [31]. Plasma concentrations of interleukin-6 (IL-6), IL-1β, and tumor necrosis factor-α (TNF-α) were measured using the Quantikine® HS ELISA Kit (R&D systems, Inc., Minneapolis, MN, USA), according to the manufacturer's instructions. The resulting color reaction was measured using the iMark™ microplate absorbance reader (Bio-Rad Laboratories, Hercules, CA, USA). The wavelength correction was set to 490 and 560 nm.

### Statistical analysis

Statistical analyses were performed with Win SPSS v21.0 (SPSS Inc., Chicago, IL). Sample-size calculation was based on the primary outcome, improvement of diarrhea during the 14-day intervention period. A sample size of 25 subjects per group with a significance level of 0.05 yielded 95 % power, therefore 100 participants were enrolled to allow for over an estimated 70 % retention rate across the study period. No stopping rules or interim analyses were planned. Differences in continuous variables between the groups were assessed using one-way analysis of variance (ANOVA) followed by the Bonferroni correction. Differences in continuous variables within a group, before and after the intervention, were analyzed using paired *t*-tests. Repeated measure ANOVA with the Bonferroni correction was also performed to examine the interactive effect between time and EN type (Time x EN type effect) on the King’s Stool scores change (%). Differences in non-continuous variables were analyzed with the chi-square test. Skewed variables were log transformed prior to statistical analysis. However, for descriptive purposes, means ± SE are presented using untransformed values. A two-tailed *P*-value <0.05 was considered statistically significant.

## Results

### Baseline characteristics of study participants

Table 2 presents baseline characteristics of the study participants completed. There were no significant differences in age, height, body weight, BMI, MAC, TSF, MAMC, BP, sex, disease history, energy intake goals, average energy intake during the intervention, or time taken to reach 100 % experimental EN feeding among the three groups.

**Table 2:** Baseline characteristics of study subjects

	Low-FODMAP (*n* = 30)	Moderate-FODMAP (*n* = 28)	High-FODMAP (*n* = 26)
Age (years)	60.1 ± 2.92	60.6 ± 3.03	62.5 ± 2.70
Female (n, %)	9 (30.0)	9 (32.1)	8 (30.8)
Weight (kg)^a^	55.4 ± 2.37	56.7 ± 2.61	55.9 ± 2.48
BMI (kg/m^2^)^a^	19.9 ± 0.68	20.6 ± 0.77	20.4 ± 0.75
MAC (cm)	25.2 ± 0.53	25.4 ± 0.70	24.2 ± 0.80
TSF (mm)	11.0 ± 0.89	12.2 ± 1.04	10.1 ± 0.88
MAMC (cm)	21.8 ± 0.50	21.5 ± 0.53	21.0 ± 0.72
Systolic BP (mmHg)	111.2 ± 2.85	116.3 ± 2.21	116.5 ± 2.76
Diastolic BP (mmHg)	74.2 ± 2.52	73.5 ± 1.52	75.0 ± 1.89
Disease history			
Hypertension	13 (43.3 %)	16 (57.1 %)	12 (46.2 %)
Diabetes^b^	5 (16.7 %)	5 (17.9 %)	6 (23.1 %)
Stroke	23 (76.7 %)	15 (53.6 %)	18 (69.2 %)
Cardiovascular disease	4 (13.3 %)	3 (10.7 %)	6 (23.1 %)
Hyperlipidemia	3 (10.0 %)	7 (25.0 %)	2 (8.00 %)
Cancer	1 (3.30 %)	1 (3.60 %)	2 (8.00 %)
Energy intake goal (kcal/day)	1273.3 ± 32.5	1310.7 ± 45.5	1269.2 ± 46.7
Average energy intake (kcal/day)	1267.2 ± 33.8	1302.6 ± 44.5	1270.5 ± 47.0
Time to reach 100 % experimental EN feeding (days)	2.57 ± 0.22	2.57 ± 0.29	2.58 ± 0.21

*BMI* body mass index, *BP* blood pressure, *MAC* midarm circumference, *MAMC* midarm muscle circumference, *TSF* triceps skin fold thickness

Means ± SE or n (%)

^a^*n* = 62

^b^Well-controlled diabetes

There were no significant differences in the baseline values among the three groups

### Distribution of major gastrointestinal symptoms before and after the intervention

There were no significant differences in the baseline distribution of gastrointestinal symptoms [normal, diarrhea, constipation, or recurrent diarrhea/constipation (D/C)] among the three EN treatment groups (Additional file 1: Table S1). ‘diarrhea*’ or ‘constipation*’ include recurrent D/C before the intervention, ‘diarrhea only’ indicates subjects who suffered from only diarrhea without recurrent D/C before the intervention, ‘constipation only’ indicates subjects who suffered from only constipation without recurrent D/C before the intervention. Gastrointestinal symptoms were daily observed for the 14 intervention days.

**Fig. 1:**
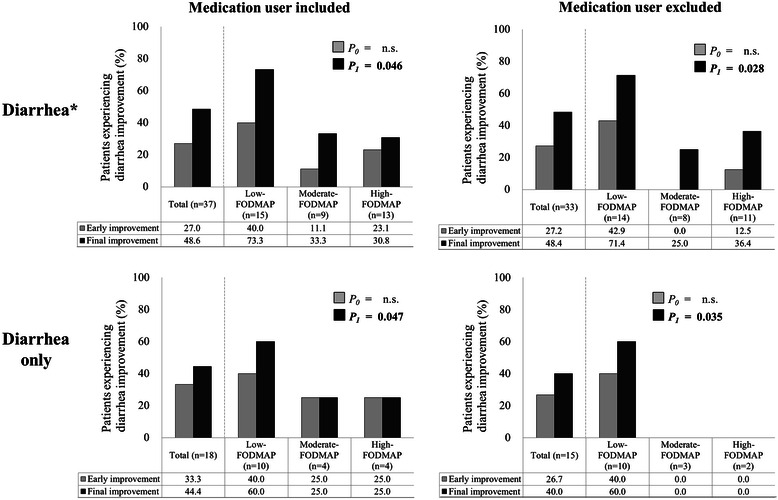
Improvement (%) of diarrhea according to EN types during the intervention. Values below the graph indicate percentages. *P*-values were obtained using the chi-square test: *P*_0_, *p*-value for differences in the improvement of diarrhea among the three EN groups within seven days from the start of intervention (early improvement); *P*_1_, *p*-value for the improvement of diarrhea or constipation among the three EN groups after the intervention (final improvement); n.s. indicates ‘statistical non-significance.’; * includes recurrent diarrhea and constipation; ‘Diarrhea only’ indicates subjects who suffered from only diarrhea without recurrent diarrhea and constipation

Figure 1 shows improvement (%) of diarrhea according to EN types during the intervention. Each subject group was also subdivided into two part ‘medication user included’ and ‘medication user excluded’. Diarrhea in the low-FODMAP group had improved significantly more than that in the moderate- or high-FODMAP groups after the intervention (final improvement). This pattern was similarly observed in each diarrhea subgroup Diarrhea improvements in the low-FODMAP group among subjects with diarrhea* including or excluding medication users were 73.3 % (*P* = 0.046) and 71.4 % (*P* = 0.028), respectively, among those with diarrhea only including or excluding medication users were 60.0 % (*P* = 0.047) and 60.0 % (*P* = 0.035), respectively. Interestingly, diarrhea improvement among the subjects with diarrhea only, but no medication users was observed only in the low-FODMAP group. Within the seven days from the start of intervention (for early improvement), diarrhea showed a trend towards improvement in the low-FODMAP group compared with the moderate- and high-FODMAP groups, but the difference did not reach statistical significance (Fig. 1).

Constipation improvements were also observed after the intervention but not significantly different among the three EN treatment groups (Additional file 1: Figure S1.A). This pattern was similarly observed in each constipation subgroup. Although constipation improvements seemed slightly higher in the high-FODMAP group than the other EN groups, the differences did not reach the statistical significances, which might be related with the small number of subjects in these groups. As shown in Additional file 1: Figure S1.B, diarrhea had improved greater in the low-FODMAP group than the moderate- or high-FODMAP groups among the subjects with recurrent D/C. This pattern was observed in each subject group including or excluding medication users (in the low-FODMAP group: 80 %, *P* = 0.017; 100 % *P* = 0.030, respectively). Constipation had improved only in the high-FODMAP group (22.2 % of ‘the medication user included’; 33.3 % of ‘the medication user excluded’), but which did not reach the statistical significance. Both diarrhea and constipation were slightly improved in the low- (20 % of ‘the medication user included’), moderate- (40 % of ‘medication user included’; 33.3 % of ‘the medication user excluded’) and high-FODMAP (11.1 % of ‘medication user included’) groups, but which were not significantly different among the groups. Participants with normal condition at baseline maintained this condition, except for one case occurrence of constipation in the moderate-FODMAP group. A few other gastrointestinal symptoms (e.g., mild abdominal pain or bloating) were observed during the intervention, but their incidence was neither high nor significantly different among the three groups (10.0 % in the low-FODMAP group, 10.7 % in the moderate-FODMAP group, and 30.8 % in the high-FODMAP group).

### Changes in King’s Stool score during intervention

**Fig. 2:**
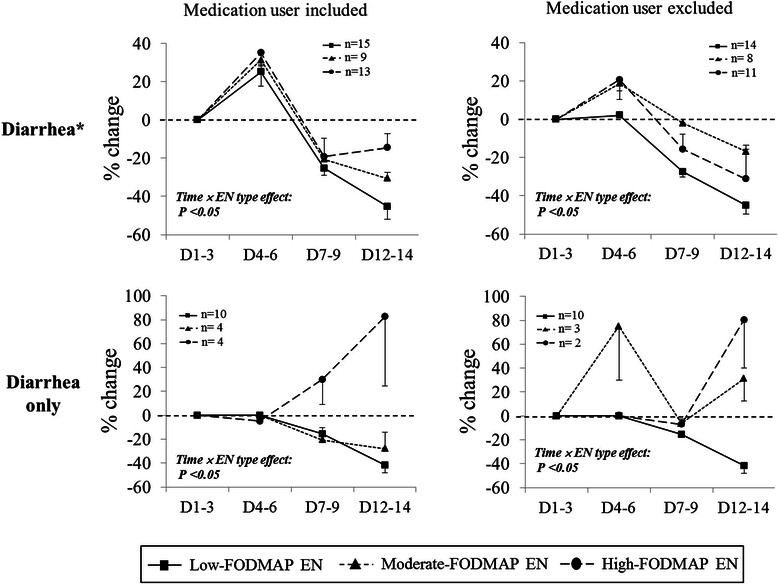
Percent changes of King’s Stool scores in diarrhea subjects according to EN types during the intervention. Values are presented as means ± S.E.; statistical significance was assessed by repeated measure ANOVA with the Bonferroni correction. *P*: *P*-values for interactive effect between time and EN type on King’s Stool scores; D1–3: adaptation period (first three days: 0-day, 1-day, 2-day); D4–6: early period (3-day, 4-day, 5-day); D7–9: mid period (6-day, 7-day, 8-day); D12–14: late period (last three days: 11-day, 12-day, 13-day)

Data for stool assessment using the King’s Stool scores chart were collected daily during the intervention period. For graphical presentation, the observation periods were categorized into four time windows: the adaptation period (D1–3, first three days: 0-day, 1-day, 2-day), the early period (D4–6: 3-day, 4-day, 5-day), the mid period (D7–9: 6-day, 7-day, 8-day), and the late period (D12–14, last three days: 11-day, 12-day, 13-day). The mean scores in each observation period were the average values calculated from the scores in each day during that period. Figure 2 presents % change of King’s Stool scores from the adaptation period (D1–3) to the early period (D4–6), the mid period (D7–9) and late period (D12–14) in the diarrhea subjects (Diarrhea* and ‘Diarrhea only’ groups). We also subdivided each subject group into two part ‘medication user included’ and ‘medication user excluded’. Repeated Measure ANOVA test was performed to examine the interactive effect between time and EN type (time × EN type effect) on the King’s Stool scores change (%). As shown in the Fig. 2, the King’s Stool scores (expressed by % changes) in the low-FODMAP group were significantly and steadily reduced during the intervention compared with the moderate- or high-FODMAP groups, which were observed in all of the diarrhea subgroups (all of *P*-values <0.05).

**Fig. 3:**
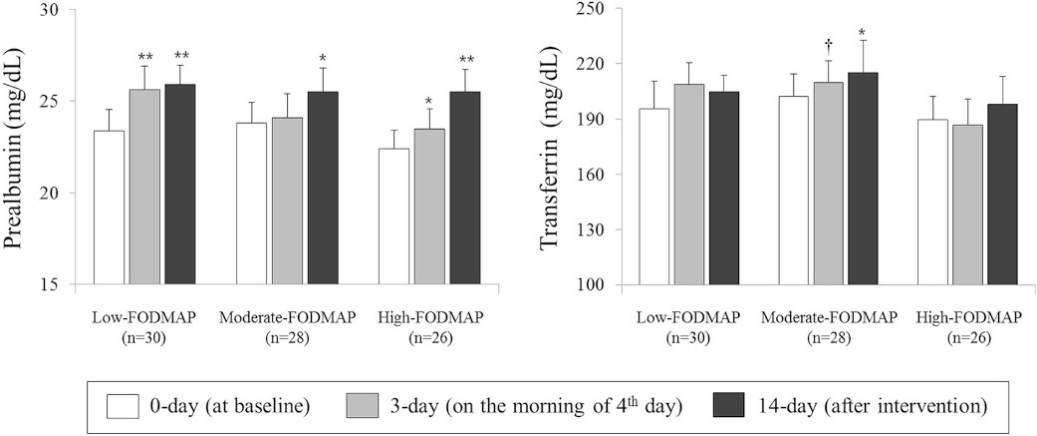
Short-term nutritional status in response to EN treatment. Values presented as means ± S.E.; statistical significance of within group differences was assessed by paired *t*-test. There were no significant differences in baseline values among the three groups. †*P* < 0.1, *P < 0.05, **P < 0.01 compared with baseline values

### Alteration of anthropometric and biochemical parameters in response to EN treatment

Anthropometric and basic biochemical parameters according to EN treatment before and after the intervention are presented in Table 3 and Additional file 1: Table S2. After the intervention, BMI significantly increased in the low- and high-FODMAP groups (*P* < 0.05 for both), and a trend toward increasing was observed in the moderate-FODMAP group (*P* < 0.10) (Table 3). After the intervention, serum levels of uric acid were significantly reduced in the low-FODMAP group compared with the moderate- and high-FODMAP groups (*P* < 0.05; Table 3). The liver function parameters serum AST (*P* < 0.01) and ALT (*P* < 0.05) increased significantly after the intervention in the moderate-FODMAP group, and the kidney function parameter serum BUN was significantly increased in the high-FODMAP group (*P* < 0.05). However, all these values were within the normal range (Table 3). Other anthropometric parameters (e.g., weight, MAC, TSF, MAMC, and BP) and basic biochemical parameters (e.g., Hb_A1C_, WBC, ALC, glucose, albumin, lipid profiles, and inflammatory markers) did not significantly change over the course of the intervention in any of the three groups (Table 3 and Additional file 1: Table S2).

**Table 3:** Anthropometric measurements, blood cell counts, and liver and kidney function markers before and after the intervention

	Low-FODMAP (*n* = 30)		Moderate-FODMAP (*n* = 28)		High-FODMAP (*n* = 26)	
	Baseline	After	Baseline	After	Baseline	After
Weight (kg)^a^	55.4 ± 2.37	55.6 ± 2.36	56.7 ± 2.61	56.3 ± 2.52	55.9 ± 2.48	55.9 ± 2.49
BMI (kg/m^2^)^a^	19.9 ± 0.68	21.3 ± 0.90^*^	20.6 ± 0.77	22.2 ± 1.07^†^	20.4 ± 0.75	22.2 ± 0.95^*^
MAC (cm)	25.2 ± 0.53	25.5 ± 0.58	25.4 ± 0.70	25.2 ± 0.77	24.2 ± 0.80	24.8 ± 0.66
TSF (mm)	11.0 ± 0.89	12.0 ± 0.82	12.2 ± 1.04	12.7 ± 1.00	10.1 ± 0.88	11.0 ± 0.92^†^
MAMC (cm)	21.8 ± 0.50	21.7 ± 0.56	21.5 ± 0.53	21.2 ± 0.66	21.0 ± 0.72	21.3 ± 0.61
Systolic BP (mmHg)	111.2 ± 2.85	110.8 ± 2.46	116.3 ± 2.21	116.8 ± 2.36	116.5 ± 2.76	117.6 ± 3.49
Diastolic BP (mmHg)	74.2 ± 2.52	74.0 ± 2.22	73.5 ± 1.52	75.7 ± 1.87	75.0 ± 1.89	74.6 ± 2.08
HbA1c (%)	5.46 ± 0.27	5.47 ± 0.34	5.52 ± 0.17	5.43 ± 0.18	5.17 ± 0.12	5.24 ± 0.13
ALC (count/μL)	1957.7 ± 132.1	1858.1 ± 83.2	1791.2 ± 123.1	1763.2 ± 121.2	1769.2 ± 137.2	1790.0 ± 147.5
WBC (×10^6^/μL)	6.62 ± 0.42	7.17 ± 0.48	7.55 ± 0.65	7.12 ± 0.53	6.90 ± 0.54	7.84 ± 0.59
AST (U/L)	20.8 ± 1.11	20.8 ± 1.50	20.3 ± 1.25	23.3 ± 1.32^**^	18.6 ± 0.95	17.8 ± 0.89
ALT (U/L)	21.8 ± 2.87	21.5 ± 2.44	16.3 ± 1.29	20.2 ± 1.59^*^	14.3 ± 1.25	15.3 ± 1.40
Creatinine (mg/dL)	0.67 ± 0.05	0.67 ± 0.04	0.70 ± 0.07	0.72 ± 0.07	0.62 ± 0.04	0.61 ± 0.04
BUN (mg/dL)	14.1 ± 1.64	13.8 ± 1.07	13.9 ± 1.40	13.5 ± 1.41	11.7 ± 0.96	12.7 ± 1.05^*^
Uric acid (mg/dL)	4.48 ± 0.27	3.99 ± 0.21^*^	5.10 ± 0.33	4.93 ± 0.27	4.74 ± 0.29	4.47 ± 0.37

*ALC* absolute lymphocyte count, *ALT* alanine aminotransferase, *AST* aspartate aminotransferase, *BMI* body mass index, *BP* blood pressure, *BUN* blood urea nitrogen, *HbA1c* glycated hemoglobin, *MAC* midarm circumference, *MAMC* midarm muscle circumference, *TSF* triceps skin fold thickness, *WBC* white blood cells

Values shown as means ± S.E

^a^ *n* = 62

†*p* < 0.1, *p < 0.05, **p < 0.01,***p < 0.001. Differences in within-group means before and after the intervention were assessed using the paired *t*-test; there were no significant differences in the baseline values among the three groups

### Alteration of short-term nutritional status in response to EN treatment

Serum albumin levels reflect nutritional status over the recent 20 days. As the intervention period in this study was only 14 days, serum prealbumin and transferrin, which reflect recent nutritional status (over the recent 2–3 days and 8–10 days, respectively), were also measured (Fig. 3). Serum levels of prealbumin increased significantly after the intervention in all three EN groups. By 3-day, serum prealbumin levels had significantly increased in the low- and high-FODMAP groups; of note, levels in the low-FODMAP group at 3-day had reached the levels at 14-day. By contrast, serum transferrin levels significantly increased after the intervention in the moderate-FODMAP group, but not in the low- or high-FODMAP groups.

**Fig. 4:**
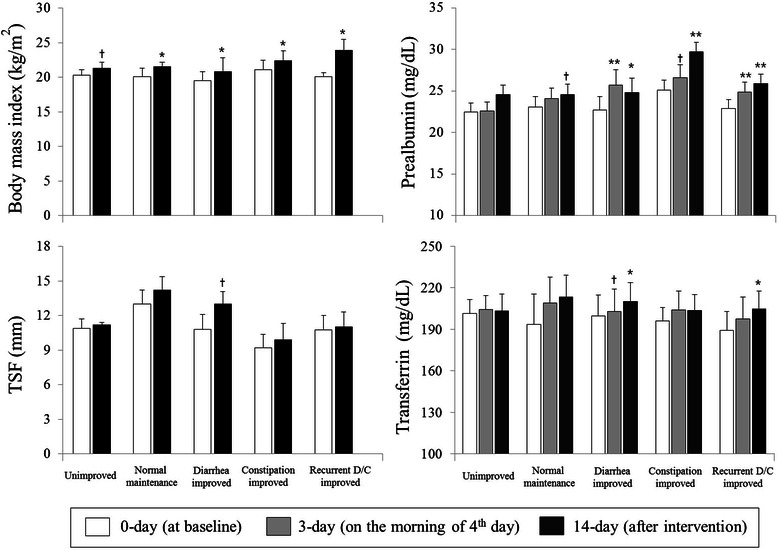
Anthropometric and nutritional status stratified by subject condition after the intervention. Values are presented as means ± S.E.; statistical significance of within group differences was assessed by paired *t*-test. There were no significant differences in baseline values among the three groups. †*P* < 0.1, *P < 0.05, **P < 0.01 compared with baseline values. Study subjects were categorized into 5 groups according to their final condition during the intervention: 1) unimproved (*n* = 29, no improvement from diarrhea or constipation), 2) normal maintenance (*n* = 22 without symptoms of diarrhea or constipation from the 23 subjects without symptoms before intervention), 3) diarrhea improved (*n* = 8 improvements from the 18 diarrhea-only subjects before the intervention), 4) constipation improved (*n* = 13 improvements from the 24 constipation-only subjects before the intervention) and 5) recurrent D/C improved (*n* = 12 improvements from the 19 recurrent diarrhea and constipation subjects before the intervention)

### Nutritional status after the intervention, stratified by subject condition

Study subjects were classified into five groups, according to their condition after the intervention (unimproved, normal-maintenance, diarrhea-improved, constipation-improved and recurrent D/C-improved: 1) unimproved (*n* = 29, no improvement from diarrhea or constipation), 2) normal maintenance (*n* = 22 without symptoms of diarrhea or constipation from the 23 subjects without symptoms before intervention), 3) diarrhea improved (*n* = 8 improvements from the 18 diarrhea-only subjects before the intervention), 4) constipation improved (*n* = 13 improvements from the 24 constipation-only subjects before the intervention) and 5) recurrent D/C improved (*n* = 12 improvements from the 19 complex condition subjects before the intervention). Additional file 1: Table S3 presents the percentage of the low-, moderate-, and high-FODMAP EN consumption in each of subgroups: 75 % of the diarrhea improved group consumed low-FODMAP EN formula. 38.5 % and 46.2 % of the recurrent D/C improved group consumed the low- and moderate-FODMAP EN formulas, respectively (Additional file 1: Table S3). As shown in Fig. 4, after the intervention, BMI significantly increased in the normal maintenance, the diarrhea improved, the constipation improved and the recurrent D/C improved groups, but not in the unimproved group. TSF showed a trend toward increasing in the diarrhea-improved group, but it did not reach statistical significance. Serum prealbumin levels in the diarrhea-improved, the constipation improved and the recurrent D/C improved groups significantly increased at 14-day. Particularly, serum preablumin levels in the diarrhea improved and the recurrent D/C improved groups were highly increased at 3-day and continued by 14-day. In the normal maintenance group, serum preablumin levels tended toward increasing at 14-day, but it did not reach statistical significance. Serum transferrin levels increased significantly in the diarrhea improved and the recurrent D/C improved groups at 14-day. By contrast, the unimproved group did not show any statistically significant changes in serum levels of prealbumin or transferrin.

## Discussion

This randomized, multicenter, double-blind clinical trial shows for the first time that a low-FODMAP formula can promptly improve diarrhea and nutritional status in patients receiving EN. In this study, diarrhea in patients receiving low-FODMAP EN was significantly improved compared with those receiving moderate- or high-FODMAP EN. King’s Stool scores in patients with diarrhea in the low-FODMAP group were also significantly reduced than those in the other two groups. In addition, serum prealbumin, a marker of short-term nutritional status, promptly improved in patients receiving low-FODMAP EN, in contrast to the other groups. When subjects were classified by condition after the intervention, markers of short-term nutritional status (prealbumin and transferrin) were significantly improved in the gastrointestinal symptom-improved groups, particularly in the diarrhea-improved group. These results support the hypothesis that low-FODMAP formula may improve or reduce the likelihood of diarrhea in patients receiving EN, leading to an improvement in nutritional status helpful for prompt recovery from illness.

Diarrhea is one of the major gastrointestional problems frequently observed in hospitalized patients receiving EN [8–10]; it may cause nutritional imbalance and consequently delay recovery [12, 17]. Many studies had tried to improve gastrointestinal problems, particularly diarrhea related to EN [2, 6, 9, 10, 14, 15, 32], but the results have been inconsistent. Recent studies have reported an association between gastrointestinal symptoms and dietary FODMAPs. Restriction of dietary FODMAPs has been reported to improve gastrointestinal symptoms, including diarrhea, in a majority of patients with IBS and IBD [20–23]. Halmos et al. suggested in his retrospective study that receiving a lower-FODMAP formula may reduce the likelihood of diarrhea among several types of formulas [8]. In our present study, patients receiving a low-FODMAP EN formula showed greater improvements in diarrhea symptoms than those who received a moderate- or high-FODMAP formula (improvement in 60.0-73.3 % of patients vs. 25.0-33.0 % and 25.0-36.4 %, respectively). After the 14-day intervention, the low-FODMAP group also showed a much greater reduction in the King’s Stool score compared with the other two groups. The mechanisms by why FODMAP restriction provides these benefits are assumed to be related to the molecular size and osmotic properties of FODMAPs [8, 33, 34]. FODMAPs are short-chain carbohydrates which have relatively smaller molecular size than non-short chain carbohydrates [8, 33, 34]. They are poorly absorbed in the small intestine and rapidly fermented by bacteria in the colon. Subsequent luminal distension may lead to secondary motility disturbance and diarrhea [23, 25]. Therefore, low-FODMAP formulas may result in less water retention within the lumen of the bowel and less gas in the intestine compared with moderate- or high-FODMAP formulas [8, 33, 34], thereby reducing gastrointestinal symptoms, particularly diarrhea.

In our study, compared with the other two groups, the low-FODMAP group showed significant improvements in serum prealbumin, a marker of short-term nutritional status at 3-day, which continued by 14-day, and in serum uric acid at 14-day. Additionally, patients with improved gastrointestinal symptoms after the intervention had greater BMIs than those who did not improve. Serum levels of prealbumin and transferrin which reflect recent nutritional status were also significantly improved in the improved gastrointestinal symptoms groups, particularly more in the diarrhea-improved group. It may be explained by that an improvement in diarrhea symptoms helps patients absorb sufficient amounts of nutrients to satisfy their nutritional requirements. These results from our randomized, double-blind, clinical trial provide the first intervention-based evidence that low-FODMAP formula improves diarrhea and nutritional status in patients receiving EN, potentially improving their nutritional status and ability to promptly recover from illness.

Our study has a few limitations. First, it is a randomized, double-blind, clinical trial where subjects were randomly assigned to one of three EN formulas. If each subject had consumed all three EN formulas via a crossover design, our conclusions would be more strongly supported. Second, long-term observation (i.e., for 2 to 6 months) may confirm whether the association between FODMAP-reduced EN feeding and improved diarrhea results in durable improvements to nutritional status and other metabolic parameters, shortening recovery time. Despite these limitations, this prospective intervention study shows that a low-FODMAP EN formula may improve diarrhea in patients receiving EN therapy, thereby improving nutritional status and potentially aiding prompt recovery from illness. Therefore, low-FODMAP EN formulas are expected to provide a useful therapeutic approach for patients with gastrointestinal symptoms, and particularly diarrhea, who are undergoing EN.

## Conclusions

The results from our randomized, double-blind, clinical trial provide the first intervention-based evidence that low-FODMAP EN improves diarrhea and nutritional status in patients receiving EN, potentially improving their nutritional status and ability to promptly recover from illness. Therefore, low-FODMAP EN formulas are expected to provide a useful therapeutic approach for patients with diarrhea undergoing EN.

## Additional file

Additional file 1: Figure S1. Improvement (%) of constipation and recurrent diarrhea/constipation according to EN types during the intervention. **Table S1.** Baseline distribution of major gastrointestinal intolerance in the study subjects. **Table S2.** Biochemical markers of nutritional status, lipid profiles, and inflammation, before and after the intervention. **Table S3.** Proportion of the low-, moderate-, and high-FODMAP EN consumption according to the subjects’ condition. (DOCX 488 kb)

## Abbreviations

ALC: Absolute lymphocyte count
ALT: Alanine aminotransferase
AST: Aspartate aminotransferase
BMI: Body mass index
BP: Blood pressure
BUN: Blood urea nitrogen
EN: Enteral nutrition
FODMAPs: Fermentable oligosaccharides, disaccharides, monosaccharides, and polyols
GI: Gastrointestinal
HbA1c: Glycated hemoglobin
HDL: High-density lipoprotein
hs-CRP: High-sensitivity C-reactive protein
IBD: Inflammatory bowel disease
IBS: Irritable bowel syndrome
IL-6: Interleukin-6
IL-β: interleukin-beta
LDL: Low-density lipoprotein
MAC: Midarm circumference
MAMC: Midarm muscle circumference
TSF: Triceps skin fold thickness
TNF-α: Tumor necrosis factor-alpha
WBC: White blood cells
