# Supplementary material for: Low-FODMAP formula improves diarrhea and nutritional status in hospitalized patients receiving enteral nutrition: a randomized, multicenter, double-blind clinical trial
Source: Nutr J. 2015 Nov 3;14:116. doi: 10.1186/s12937-015-0106-0 (PMC4632275; doi:10.1186/s12937-015-0106-0)
Supplement: Additional file 1: Figure S1. — Improvement (%) of constipation and recurrent diarrhea/constipation according to EN types during the intervention. Table S1. Baseline distribution of major gastrointestinal intolerance in the study subjects. Table S2. Biochemical markers of nutritional status, lipid profiles, and inflammation, before and after the intervention. Table S3. Proportion of the low-, moderate-, and high-FODMAP EN consumption according to the subjects’ condition. (DOCX 488 kb) [file 12937_2015_106_MOESM1_ESM.docx]

**
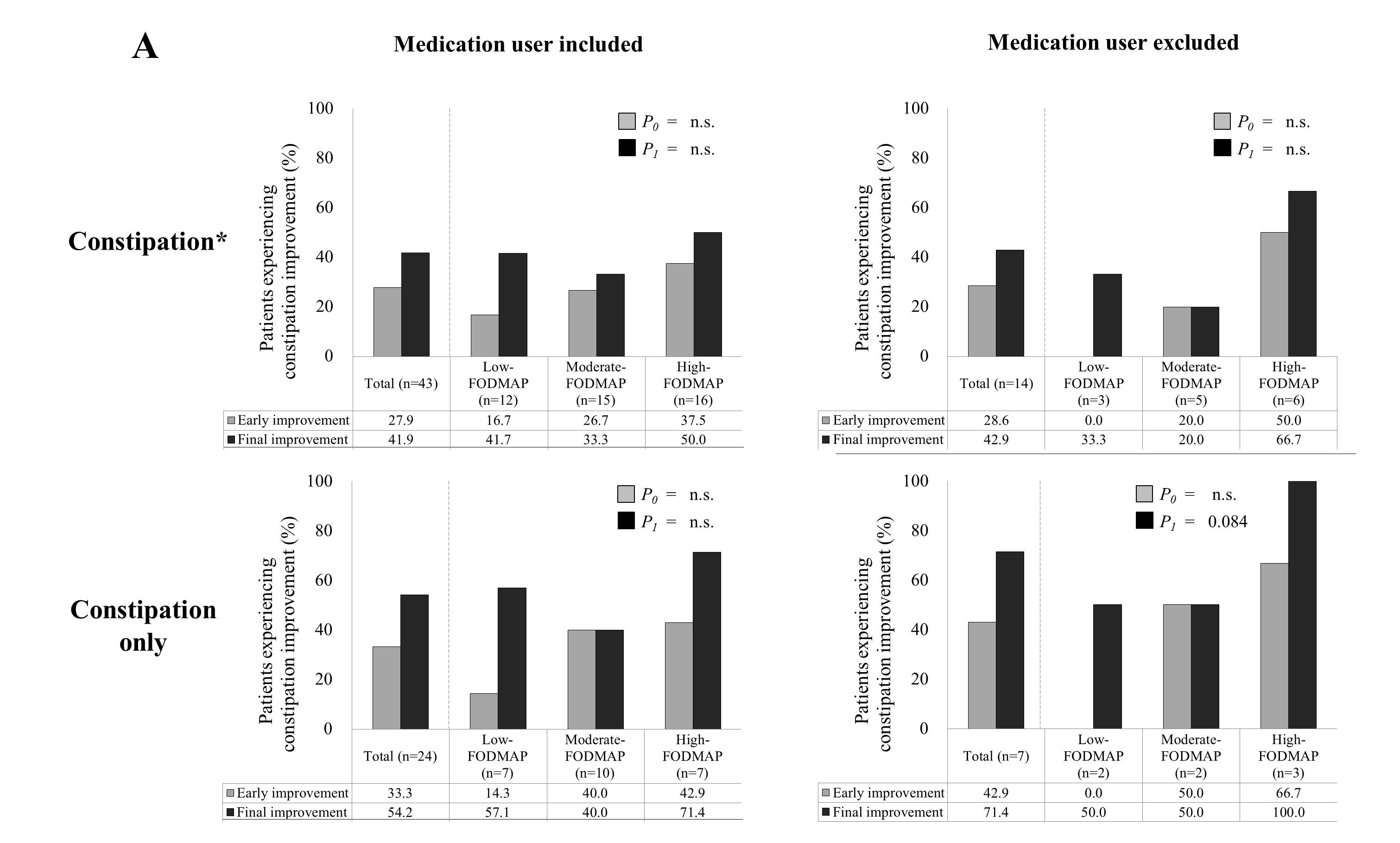
**

**
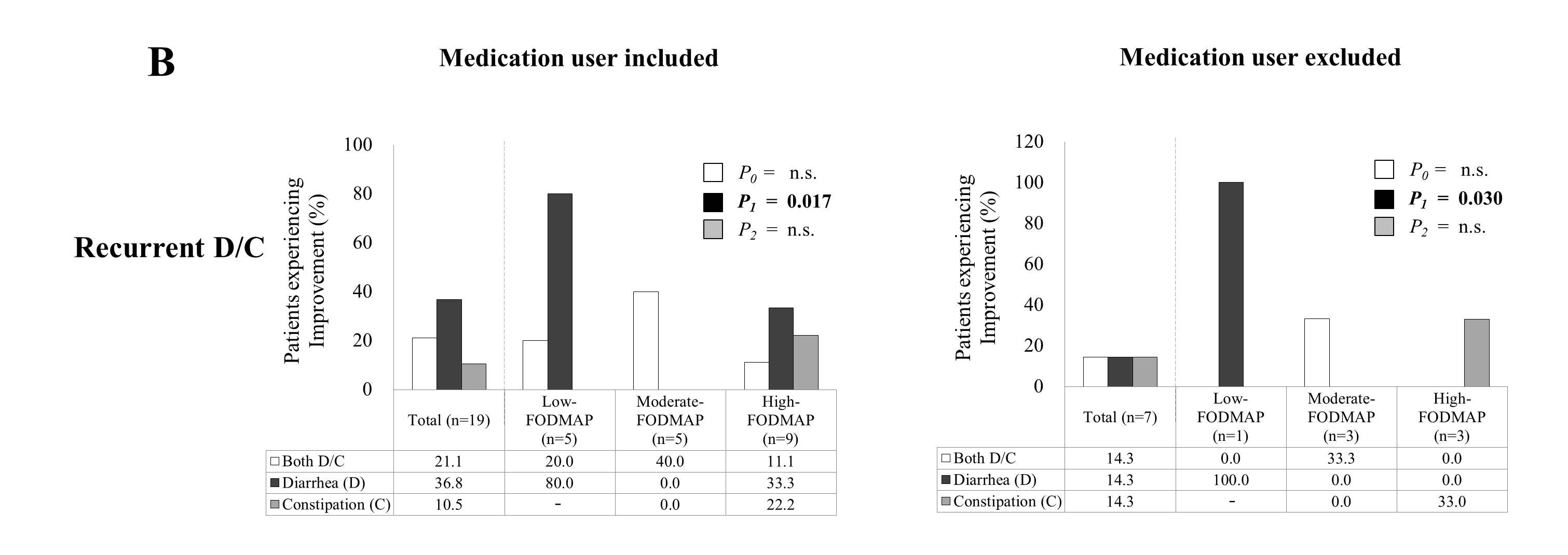
**

**Supplementary Figure 1. Improvement (%) of constipation and recurrent diarrhea/constipation according to EN types during the intervention.** A) improvement (%) of constipation according to EN types, B) improvement (%) of recurrent D/C according to EN types; Values below the graph indicate percentages. *P*-values were obtained using the chi-square test: *P*_0_, *p*-value for differences in the improvement of diarrhea or constipation among the three EN groups within seven days from the start of intervention (early improvement); *P*_1_, *p*-value for the improvement of diarrhea or constipation among the three EN groups after the intervention (final improvement); n.s. indicates ‘statistical non-significance.’; * includes recurrent diarrhea and constipation; ‘Constipation only’ indicates subjects who suffered from only constipation without recurrent diarrhea and constipation; ‘Recurrent D/C’ indicates subjects who suffered from recurrent diarrhea and constipation.

Supplementary Table A1. Baseline distribution of major gastrointestinal intolerance in the study subjects

| n (%) | | Total  n=84 |  | | Low-FODMAP  n=30 | | Moderate- FODMAP  n=28 | High- FODMAP  n=26 | *P* |
| --- | --- | --- | --- | --- | --- | --- | --- | --- | --- |
| **Normal** | | **23 (27.4)** |  | | **8 (26.7)** | | **9 (32.1)** | **6 (23.1)** | **n.s.** |
| Diarrhea* | | 37 (44.0) |  | | 15 (50.0) | | 9 (32.1) | 13 (50.0) | n.s. |
|  | Anti-diarrhea medication user | 4 (10.8) |  | | 1 (6.7) | | 1 (11.1) | 2 (15.4) | n.s. |
| **Diarrhea only** | | **18 (21.4)** |  | | **10 (33.3)** | | **4 (14.3)** | **4 (15.4)** | **n.s.** |
| Constipation* | | 43 (51.2) |  | | 12 (40.0) | | 15 (53.6) | 16 (61.5) | n.s. |
|  | Enema user | 17 (39.5) |  | | | 5 (41.7) | 6 (40) | 6 (37.5) | n.s. |
|  | Stool softener user | 15 (34.9) |  | | | 6 (50.0) | 5 (53.3) | 4 (25.0) | n.s. |
| **Constipation only** | | **24 (28.6)** |  | | | **7 (23.3)** | **10 (35.7)** | **7 (26.9)** | **n.s** |
| **Recurrent D/C^1^** | | **19 (22.6)** |  | | | **5 (16.7)** | **5 (17.9)** | **9 (34.6)** | **n.s.** |
|  | Anti-diarrhea medication user | 1 (5.30) |  | | 1 (20.0) | | 0 (0.00) | 0 (0.00) | n.s. |
|  | Enema user | 6 (31.6) |  | 2 (40.0) | | | 1 (20.0) | 5 (55.6) | n.s. |
|  | Stool softener user | 6 (31.6) |  | 4 (80.0) | | | 1 (20.0) | 1 (11.1) | n.s. |
| **Sum total** | | **84 (100)** |  | **30 (100)** | | | **28 (100)** | **26 (100)** | **-** |

Presented as numbers (%), with statistical significance assessed using the chi-square test.

^1^ Recurrent D/C: Recurrent diarrhea and constipation (complex condition); * include recurrent D/C

Supplementary Table A2. Biochemical markers of nutritional status, lipid profiles, and inflammation, before and after the intervention

|  |  | Low-FODMAP (n=30) | | | | | |  | Moderate-FODMAP (n=28) | | | | | |  | High-FODMAP (n=26) | | | | | |  |
| --- | --- | --- | --- | --- | --- | --- | --- | --- | --- | --- | --- | --- | --- | --- | --- | --- | --- | --- | --- | --- | --- | --- |
|  |  | Baseline | | | After | | |  | Baseline | | | After | | |  | Baseline | | | After | | |  |
| Albumin (g/dL) |  | 3.81 | ± | 0.12 | 3.83 | ± | 0.08 |  | 3.71 | ± | 0.07 | 3.76 | ± | 0.07 |  | 3.74 | ± | 0.06 | 3.77 | ± | 0.06 |  |
| Glucose (mg/dL) |  | 85.1 | ± | 7.68 | 83.5 | ± | 6.83 |  | 91.0 | ± | 4.43 | 86.85 | ± | 5.02 |  | 84.5 | ± | 3.43 | 83.2 | ± | 3.56 |  |
| Triglycerides (mg/dL) |  | 131.7 | ± | 14.1 | 134.2 | ± | 15.4 |  | 136.1 | ± | 16.0 | 164.5 | ± | 18.6^**^ |  | 126.5 | ± | 10.8 | 140.0 | ± | 13.5 |  |
| Total cholesterol (mg/dL) |  | 152.3 | ± | 7.62 | 160.6 | ± | 6.07 |  | 161.3 | ± | 8.00 | 169.4 | ± | 9.35^*^ |  | 167.3 | ± | 6.34 | 170.2 | ± | 6.96 |  |
| LDL cholesterol (mg/dL) |  | 84.2 | ± | 5.67 | 90.7 | ± | 5.12 |  | 89.7 | ± | 5.93 | 93.8 | ± | 6.66 |  | 99.9 | ± | 5.23 | 103.2 | ± | 5.42 |  |
| HDL cholesterol (mg/dL) |  | 46.1 | ± | 2.87 | 47.3 | ± | 2.70 |  | 44.1 | ± | 2.35 | 44.6 | ± | 1.76 |  | 44.2 | ± | 3.01 | 44.0 | ± | 2.76 |  |
| hs-CRP (mg/dL) |  | 5.51 | ± | 1.38 | 4.37 | ± | 0.97 |  | 5.66 | ± | 1.32 | 7.75 | ± | 2.50 |  | 6.89 | ± | 2.30 | 5.12 | ± | 1.45 |  |
| IL-6 (pg/mL) |  | 3.94 | ± | 1.16 | 3.98 | ± | 0.98 |  | 3.81 | ± | 1.03 | 3.89 | ± | 0.79 |  | 6.28 | ± | 2.34 | 4.65 | ± | 1.52 |  |
| TNF-α (pg/mL) |  | 2.21 | ± | 0.35 | 2.22 | ± | 0.33 |  | 1.86 | ± | 0.26 | 1.85 | ± | 0.20 |  | 2.22 | ± | 0.57 | 2.15 | ± | 0.45 |  |
| IL-β (pg/mL) |  | 0.40 | ± | 0.12 | 0.46 | ± | 0.13 |  | 0.14 | ± | 0.05 | 0.14 | ± | 0.04 |  | 0.34 | ± | 0.11 | 0.92 | ± | 0.33 |  |

Values shown as means ± SE.

†p<0.1, *p<0.05, **p<0.01 before and after the intervention in each group; there were no significant differences in baseline values among the three groups. HDL: high-density lipoprotein, hs-CRP: high-sensitivity C-reactive protein, IL-6: interleukin-6, IL-β: interleukin beta, LDL: low-density lipoprotein, TNF-α: tumor necrosis factor-alpha

Supplementary Table A3. Proportion of the low-, moderate-, and high-FODMAP EN consumption according to the subjects’ condition

| Consumption (%) | Unimproved | Normal maintenance | Diarrhea improved | Constipation improved | Recurrent D/C improved |
| --- | --- | --- | --- | --- | --- |
| Low-FODMAP | 25.0 | 36.4 | **75.0** | 30.8 | **38.5** |
| Moderate-FODMAP | 28.6 | 27.3 | 12.5 | 38.5 | **46.2** |
| High-FODMAP | 46.4 | 36.4 | 12.5 | 30.8 | 15.4 |
| Total sum (%) | 100 | 100 | 100 | 100 | 100 |

presented as percent (% )
